# Quantifying the Impact of Multiple Twin-Screw Extrusion Cycles on Discontinuous Carbon Fiber Composites

**DOI:** 10.3390/polym18212634

**Published:** 2026-10-23

**Authors:** Roo Walker, Matthew Korey, Amber M. Hubbard, Daniel Webb, Evan J. Holt, Ally Collier, Katie Copenhaver, S. Michael Kilbey, Soydan Ozcan, Chad Duty

**Affiliations:** 1 Bredesen Center for Interdisciplinary Research and Graduate Education, The University of Tennessee, Knoxville, TN 37996, USAcduty@utk.edu (C.D.); 2 Oak Ridge National Laboratory, Oak Ridge, TN 37831, USA; 3 Department of Chemistry, The University of Tennessee, Knoxville, TN 37996, USA; 4 Department of Mechanical and Aerospace Engineering, The University of Tennessee, Knoxville, TN 37996, USA

**Keywords:** carbon-fiber-reinforced thermoplastics, recyclability, pilot-scale manufacturing, fiber length, composite rheology

## Abstract

To enable re-use, recycled fiber-filled thermoplastics may require intermediate processing, such as twin-screw compounding. These intermediate processes can introduce additional processing-induced degradation and performance variability. This study evaluated the recyclability of carbon-fiber-reinforced acrylonitrile butadiene styrene (CF-ABS) through ten consecutive pilot-scale extrusion cycles to determine the primary degradation mechanism as a function of lifetime and to quantify the impact on mechanical performance. Chemical analysis revealed that the ABS matrix remained chemically robust throughout repeated extrusion cycles, whereas optical microscopy showed that fiber breakage was significant. Fiber length exponentially decayed with repeated cycles, with the decay rate stabilizing between the 3rd and 5th cycle, resulting in a 62% reduction in weight-averaged fiber length from C0 to C10. The reduction in fiber length was found to govern both the rheological and mechanical behavior of the composite material. Viscosity and storage modulus decreased with decreasing fiber length while tan(δ) and elongation at break increased, shifting toward values characteristic of neat ABS and indicating a transition from fiber-dominant to matrix-dominant behavior. The tensile strength decreased 35% after the 10th cycle, and its decay behavior correlated with the decay rate of the measured fiber length. This correlation enabled the development of a simple analytical model to predict tensile strength, which predicted tensile strength more accurately than the Kelly–Tyson model (RMSE of 38 reduced to 3.5) and required fewer experimentally derived inputs. Overall, this study demonstrated that fiber breakage, rather than chemical degradation of the ABS matrix, dictates performance of recycled CF-ABS.

## 1. Introduction

As the demand for sustainable and recyclable materials increases, thermoplastic composites, such as short carbon-fiber-reinforced plastics (CFRPs), are valuable materials for a variety of industries and applications due to their versatility, recyclability, and their ability to achieve more advantageous material properties, such as high strength to weight ratios [1,2,3]. CFRPs have seen specific success in industries like automotive and aerospace, partly due to the adoption of these materials for the rapid development of tools, fixtures, and molds [4,5]. Short discontinuous fiber composites can utilize a number of synthetic or natural fibers as reinforcement, but carbon fiber (CF) is most often used in applications where high stiffness parts are necessary. However, as the demand for composites continues to increase, financial and environmental impacts must also be considered. CF as a reinforcing filler, while an overall smaller market share compared to glass fiber, represents a much higher commercial value (~10×) and energy demand (~14×) [6]. Due to the high cost, energy demand during production, and commercial value, reclaiming and recycling CFRPs are essential in developing a more robust and sustainable supply chain for CFRP materials [7].

Mechanical recycling, the process where parts or scraps are size reduced by mechanical processes, such as shredding and granulation, has been demonstrated as a useful recycling pathway to recover these materials. However, waste streams targeted for recycling are often heterogeneous, having a wide range of shapes and sizes, such as granulate recovered from mechanical shredding [8,9] or flake-like particulates from machining waste [10,11]. While some efforts have explored the direct use of granulated recycled CFRP materials [8,12] into manufacturing processes, these methods have yet to see widespread industrial adoption. Until manufacturing technologies are capable of processing mixed particle size recyclate into high-quality parts, direct re-use of mixed-size recyclate can result in defects (e.g., poor consolidation) in extrusion or injection-based manufacturing processes [10,13,14]. To address the concern of material shape irregularity, re-extrusion and pelletization of recyclate through intermediary compounding processes, like twin-screw extrusion (TSE), may be introduced as an additional step to the remanufacturing pathway in order to pelletize material into a more homogenously shaped feedstock [15]. Therefore, it is important to consider how the re-extrusion and pelletization steps add to the overall degradation of CFRP materials during recycling prior to further manufacturing processes. Intermediary TSE processes have the potential to introduce thermomechanical degradation to composites due to the high shear rates and thermal energy used, potentially negatively impacting the performance of the resulting part. This is especially important to consider across multiple material lifetimes and on a pilot scale.

The adoption of recycled CFRPs has been limited due to concerns of performance limitations stemming from the potential for matrix and fiber phase degradation [16]. A material stream of interest for recycling and remanufacturing is end-of-life (EOL) CFRP parts and manufacturing waste recovered from large format additive manufacturing (LFAM). CFRPs, such as carbon fiber acrylonitrile butadiene styrene (CF-ABS), have been instrumental in the development of LFAM technologies for composite mold and tooling applications [17,18], with this industry having an anticipated cumulative annual growth rate of 8.4% through 2033 [19]. With the expected increase in the volume of EOL composites as a result of the growing LFAM market, it is essential to investigate the impact of remanufacturing pathways on material degradation and performance. This study focused on CF-ABS with 20 wt% of short carbon fiber due to its high value nature for subsequent injection and extrusion processes. Previous studies have shown that there is minimal chemical degradation in unfilled ABS after multiple extrusion cycles at 230–240 °C [20,21,22]. However, there is some data to suggest that the polybutadiene (PB) phase can undergo crosslinking reactions during recycling and remanufacturing, though this was observed without significant changes in the mechanical properties [23]. Similarly, previous characterization of recycled 20 wt% CF-ABS recovered from machining waste has shown minimal impacts on the matrix phase after recycling and remanufacturing [10]. Fiber phase degradation [24,25,26,27], particularly fiber breakage during TSE, is a critical concern for recycled CFRP materials as fiber length aids in the load transfer process in composites. Fiber length due to breakage in processing has been shown to follow exponential decay behavior [28], aligning with Tucker’s model for fiber length conservation [25,29], which concluded that there is an unbreakable or terminal length achieved after multiple screw-based processing steps. Understanding the rate of fiber length degradation with respect to the number of TSE re-extrusion cycles is essential for accurately predicting mechanical properties using micromechanical models, such as the Kelly–Tyson model, and for developing predictive analytical models for recycled CFRP performance.

Therefore, in order to de-risk the industrialization of recycling CF-ABS for extrusion and injection molding applications, it is critical to understand the mechanism and rate of degradation imparted on the matrix and fiber phases during intermediate thermomechanical processes at a pilot-scale. As recycled composite materials enter manufacturing supply chains, addressing the literature gap between lab/bench-scale and pilot-scale performance is necessary for material qualification, material readiness, and process readiness [16,30]. This study provides a pilot-scale cycle-to-cycle dataset for CF-ABS subjected to repeated TSE and pelletization, where an initial 500 lb of material was processed through ten TSE re-extrusion cycles. The resulting re-extruded material was then assessed for its primary degradation mechanism, processing characteristics, and mechanical performance. In addition, this study also introduced a simple analytical model for the prediction of tensile strength with respect to the fiber length degradation.

## 2. Materials and Methods

Five-hundred pounds of CF-ABS with 20 wt% carbon fiber (Electrafil 1501 3DP, Techmer PM, Clinton, TN, USA) was used as the pristine (C0) material for this study and was used as received. The pristine material was extruded and pelletized 10 times, as detailed below, comprising sample sets denoted as cycle 1 (C1) through cycle 10 (C10).

### 2.1. Twin-Screw Extrusion and Injection Molding

Twin-screw extrusion was performed using a Leistritz ZSE-27 MAXX (Allendale, NJ, USA) to re-extrude the CF-ABS material. The extruder had co-rotating intermeshing screws, a 40:1 L/D ratio, and barrel temperature zones were set according to the material’s thermal stability (Table S1, Figure S1), with temperatures increasing progressively along the extrusion profile, peaking before the die block to pull away any melt gases. An average throughput of 18 kg/h was maintained to regulate shear and material flow. The residence time was approximately 120 s and the temperature of the melt at the die was observed to be 234 °C. Extruded filaments through a circular die (3.5 mm diameter) were fed into a pelletization system and chopped into cylindrical pellets of average length ~4 mm. After each re-extrusion cycle, approximately 30 lbs. of pellets were set aside for further manufacturing and material property investigation.

Pellets from selected cycles: C0, C1, C3, C5, and C10 were molded into testing specimens on a BOY 35 EVV injection molding system (BOY Machines, Inc., Exton, PA, USA) with an L/D ratio of 18.6:1. Injection parameters are listed in Table S2. A minimum of 5 samples per geometry were manufactured.

### 2.2. Gel Permeation Chromatography (GPC)

The molecular weight distributions of C0–C10 pellets were measured using GPC. Neat ABS pellets were utilized in this experiment as a comparison material. Each of the samples was dissolved in tetrahydrofuran (THF) with magnetic stirring. The resulting solutions were then filtered to remove fiber from the solutions and distributed into high-performance liquid chromatography (HPLC) vials for automated injection into the GPC. The molecular weight and dispersity of each sample was determined at room temperature using an Agilent 1260 Infinity II GPC system (Agilent, Santa Clara, CA, USA) that uses THF as the mobile phase at a flow rate of 1.0 mL/min. For these measurements, an Optilab T-rEX differential refractometer (Wyatt Technologies, Santa Barbara, CA, USA) was used for detection, and molecular weight values were determined via conventional calibration analysis using polystyrene standards over the range of 850 g/mol to 364,000 g/mol. Molecular weight data for all 10 cycles are presented in Figure S1.

### 2.3. Attenuated Total Reflection Fourier Transform Infrared Spectroscopy

Attenuated Total Reflectance Fourier Transform Infrared Spectroscopy (ATR-FTIR) was performed on all samples (C0–C10) to determine if there were changes in the chemical structure of the materials as a result of re-extrusion. ATR-FTIR spectra were obtained using a Nicolet iS50 FTIR Spectrometer (Thermo Scientific, Madison, WI, USA) with a diamond crystal for ATR. Samples in the form of pellets were deposited directly onto the diamond crystal for analysis. The instrument resolution was 4 cm^−1^ and each sample was scanned for 64 scans. Baseline measurements of the environment were collected and removed from the spectra. The observed spectra were not normalized to prevent loss of data resolution in the fingerprint region.

### 2.4. Thermal Analysis

A TA Instruments Discovery series differential scanning calorimeter (DSC) 2500 (TA Instruments, New Castle, DE, USA) was used to collect thermal properties on materials from each cycle. The samples were tested with a heat–cool–heat ramp between 0 °C and 190 °C at a temperature ramp rate of 10 °C/min in an air environment. The glass transition temperature (T_g_) was calculated using the inflection point from the transition observed from the second heat ramp. Triplicates were run for each cycle to ensure statistical significance.

### 2.5. Thermogravimetric Analysis

A TA Instruments Discovery series thermogravimetric analyzer (TGA) 5500 (TA Instruments, New Castle, DE, USA) was used to observe the degradation behavior and the residual fiber content of all materials. A temperature ramp from 30 °C to 600 °C with a heating rate of 10 °C/min was completed for all samples in both air and an ultra-high purity argon environment. Triplicates were run for all cycles to ensure statistical significance. The decomposition temperature was measured at 2 wt% and 5 wt% mass loss to assess the thermal stability, and these temperatures are denoted as T_2%_ and T_5%_, respectively. The final weight percent of each sample type at 600 °C was recorded to quantify the fiber content.

### 2.6. Fiber Length Analysis

Fiber imaging was completed where acetone-aided digestion was used to isolate the CF from the ABS polymer matrix. For the acetone digestion, ~0.5 g of material (~3 pellets) was added to 50 mL of acetone. The pellets were chosen randomly from the ~30 lbs of materials collected after TSE cycle. This solution was mixed using bath sonication for two 99 min cycles at room temperature water. Once the material was dissolved, approximately 20 mL of acetone was added to the CF-ABS-acetone solutions. The solutions were then applied to microscopy slides and given time for the acetone to evaporate from the slide surface and imaged on a Keyence Optical Microscope (Keyence Corp. of America, Itasca, IL, USA) with 50× total magnification. One image per slide was taken using the stitching function, with a minimum of two images taken per cycle. The images were processed using the Fiji processing package for ImageJ 1.54P [31]. The fibers were then measured, excluding any unclear, incompletely imaged, or clumped fibers. A minimum of 1000 fibers were imaged and measured per cycle. The results were analyzed for statistical significance (*p* < 0.05) and their distributions were observed using a log-normal distribution analysis [32]. To compute the average fiber length and standard deviations, fibers were grouped into sub-samples of 200 fibers, with each cycle having a minimum of 5 sub-groups. These sub-samples were grouped arbitrarily and did not rely on any size or length characteristics to define the groups.

### 2.7. Rheological Characterization

The complex viscosities of C0–C10 samples were measured with a TA Instruments Discovery Hybrid Rheometer-2 (DHR2) (TA Instruments, New Castle, DE, USA). Rheological testing was done with a 25 mm diameter parallel plate geometry at 235 °C, the approximate temperature of the polymer melt at the exit of the TSE (Table S1). Materials were tested using an oscillatory frequency sweep in air, with a frequency range of 0.1–628 radians per second, a gap height of 1.5 mm, and an applied strain of 0.1%. Three sweeps were performed on each material to ensure statistical significance.

### 2.8. Dynamic Mechanical Analysis (DMA)

DMA was performed on a TA Instruments DMA 850 (TA Instruments, New Castle, DE, USA) for all injection-molded parts using the dual cantilever fixture. The sample dimensions for all samples were approximately 60 mm × 12 mm × 2 mm. Initially, strain and frequency sweeps were performed at room temperature to determine the amplitude and frequency limitations of the linear viscoelastic regime. Subsequently, temperature sweeps were performed from room temperature to 175 °C at a temperature ramp rate of 2 °C min^−1^ under a strain of 0.01% and a frequency of 1 Hz. A minimum of 3 samples were tested for C0, C1, C3, C5, and C10 to ensure statistical significance.

### 2.9. Mechanical Characterization

Young’s elastic modulus, ultimate tensile strength (UTS), elongation at break (EB), and fracture toughness of the injection-molded samples were measured via tensile testing to determine the influence of the re-extrusion process on mechanical performance. Samples C0, C1, C3, C5, and C10 were chosen as cycles of primary interest for mechanical testing. The tensile specimens were molded to Type 1 size specifications per ASTM D638 [33] standards. The tensile testing was completed at a rate of 5 mm/min on an MTS Criterion Series, Model 45 (MTS Systems, Eden Prairie, MN, USA) with a 100 kN load cell. Extension was measured using an MTS LX 500 Laser Extensometer (MTS Systems, Eden Prairie, MN, USA). A minimum of three samples were tested for each cycle. Fracture toughness was assessed by calculating the area under the stress–strain curves produced from tensile testing. Two data points for C0 were excluded due to breaking outside of the extensometer gauge length; however, enough data was retained for statistical significance evaluation.

### 2.10. Curve Fitting and Statistical Analysis

Data were analyzed for statistical significance using a Student’s *t*-test. An alpha of 0.05 was used to determine significance and, if necessary, assessments at an alpha level of 0.01 were performed. To determine the degradation rate of the material and to analyze the resulting behaviors with respect to the number of re-extrusion cycles, JMP [34] was used to assess the non-linear curve trends and determine the confidence intervals for the corresponding behavior. JMP Pro 17 was also used to assess the curve-fit of the analytical trend predictions and to produce model test statistics.

## 3. Results and Discussion

### 3.1. Matrix Phase Degradation

**Figure 1:**
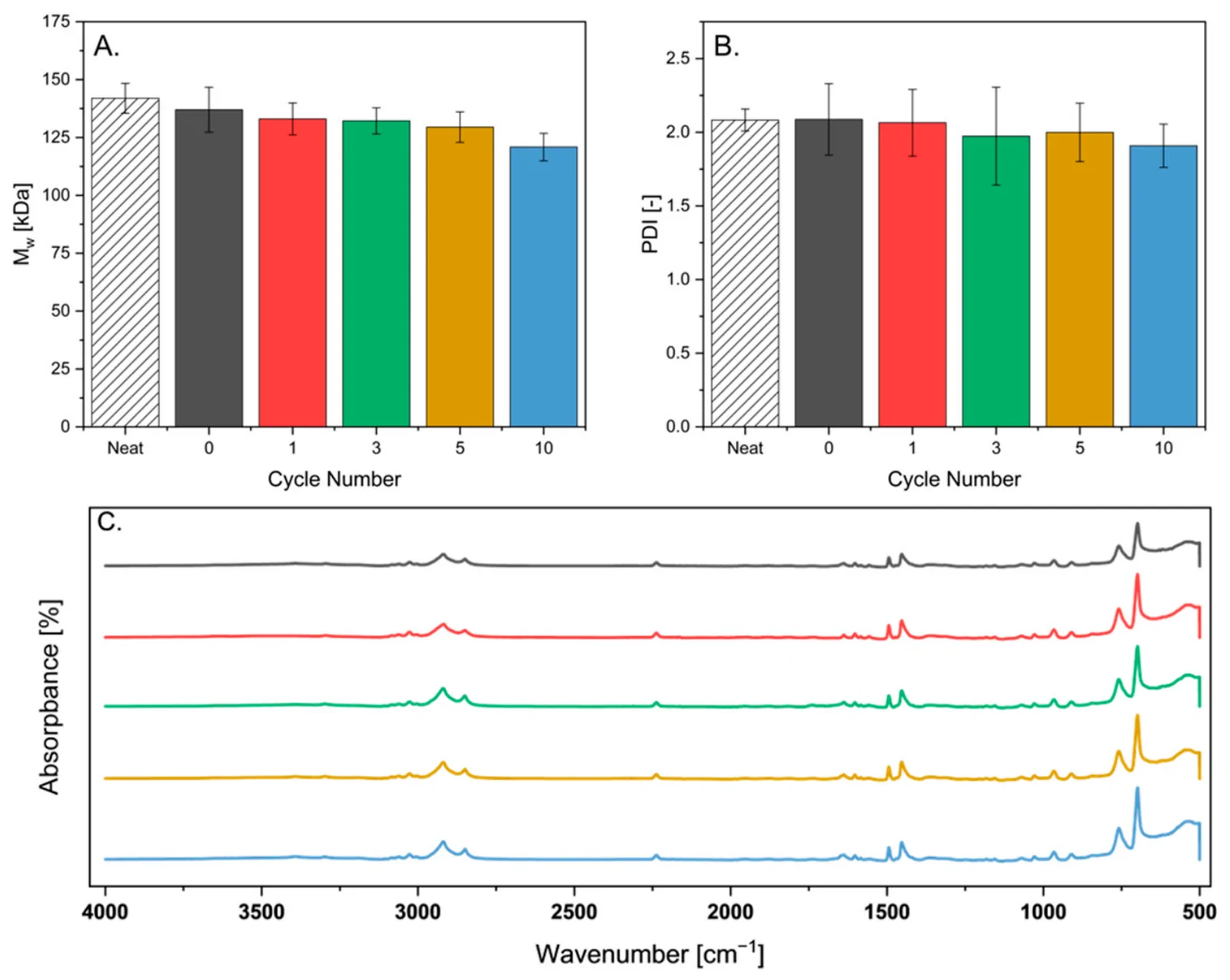
GPC results of C0–C10 where both (**A**) M_w_ and (**B**) Ð are reported; the results indicate that there is no substantial change in molecular weight after 10 thermomechanical re-extrusion cycles. When assessing the potential for PB crosslinking, collected FTIR spectra (**C**) shows no significant change in chemical structure after 10 re-extrusion cycles. The error bars in (**A**,**B**) represent the standard deviation of measurements.

Previous research [23] has indicated potential for ABS to undergo degrative thermo-oxidation after multiple re-extrusion cycles, so it was necessary to investigate the matrix phase for evidence of chemical degradation that could contribute to decreasing material performance. In ABS, thermo-oxidation can lead to chain scission reactions, crosslinking of the polybutadiene (PB) phase, and loss of small molecular weight products [22,35]. Chain scission reactions at elevated temperatures result in a reduction in molecular weight of the styrene–acrylonitrile (SAN) phase, and reductions in molecular weight below the weight of entanglement can negatively impact properties like UTS and T_g_ [36,37]. To characterize the potential for molecular weight degradation, GPC was utilized to assess the weight-average molecular weight (M_w_) and dispersity (Ð) after each re-extrusion cycle (Figure 1 and Figure S2). There was no significant (*p* > 0.05) change in recorded Ð in C1–C10 in comparison to C0. M_w_ remained statistically unchanged from C1–C9. Although C10 exhibited a slight decrease relative to C0, the overall averages across cycles were relatively consistent and did not exhibit a trend indicative of molecular weight degradation (cf. Figure S1). These results demonstrate minimal degradation of the ABS matrix due to multiple re-extrusion cycles.

Under certain thermomechanical conditions, polybutadiene in ABS could undergo crosslinking, potentially leading to THF insoluble fractions that may not be detected by GPC [38]. Although the processing temperatures in this study were below the thermal degradation temperatures for ABS, FTIR was performed on pellets collected from each cycle to verify that there were no chemical changes associated with polybutadiene crosslinking (Figure 1C). ABS is characterized by the nitrile (C≡N) signal at 2238 cm^−1^ and the aromatic C-H signal at 760 cm^−1^. The C-H aromatic and alkene signals can be seen from 3100 to 3000 cm^−1^ while the C-H aliphatic signals are located just below at 2916 and 2851 cm^−1^. There was no significant change in the collected spectra across all samples. If crosslinking was occurring, then it is expected that the absorbance bands at 967 and 908 cm^−1^ would decrease, indicating a chemical change in the trans C=C unsaturation (vinyl) in polybutadiene, and the 1,2-butadiene terminal vinyl C–H band, respectively. Due to there being no notable change in the collected spectra as well as the values of degradation temperature, which are significantly higher than the processing temperature used here (cf. Table S4), it is believed that there is minimal evidence that crosslinking or other chemical degradation processes occurred in ABS, even after multiple re-extrusion cycles. These results are similar to those presented in Bai et al. [21], who also observed minimal changes in chemical structure after re-extrusion at similar temperatures. Further, the T_g_ of the re-extruded CF-ABS samples was observed using DSC and DMA (Figure S3 and Table S3) and compared to C0. When evaluating T_g_ by DSC, a slight increase was identified; however, the observed shift was ~2 °C relative to C0. Given the breadth of the glass transition region and its sensitivity to heating rate and analysis method [39], this difference falls within expected experimental variability. When T_g_ was evaluated by DMA (Table S3), there was no significant difference identified (*p* > 0.05). Therefore, it is postulated that matrix phase degradation was minimal and will have minimal impact on the material performance.

### 3.2. Fiber-Phase Degradation

CFRPs are highly dependent on both fiber weight content and fiber length in order to achieve maximum load transfer and superior mechanical properties [40,41,42]. Because of this, it was necessary to track the degradation of the fiber phase as a function of the number of re-extrusion cycles. During the re-extrusion process, small amounts of dust were observed in the material hopper. Therefore, it was important to ensure that there was no significant loss of fiber weight content with subsequent re-extrusion cycles, so TGA was performed in an inert environment on pellets from each cycle and the amount of residual material was recorded at 600 °C (Table S4). No significant difference was found in the residual fiber content after 10 re-extrusion cycles, indicating that changes in mechanical properties are unlikely to be due to any changes in fiber content.

Fiber breakage is widely recognized as a key challenge in the utilization of recycled composite feedstocks. Fiber breakage can occur in TSE re-extrusion processes because of fiber–fiber, fiber–matrix, and fiber–machine interactions [43]. To investigate fiber length degradation and the rate at which it occurs after multiple re-extrusion cycles, the number-average (L_n_) and the weighted-average (L_w_) fiber lengths were measured (cf. Equations (S1) and (S2)). Similar to assessing the average molecular weight of a polymer, the number- and weight-average fiber lengths capture different aspects of the filler’s size and contribution to resulting properties. L_n_ is important to observe as it serves as a more representative value for the actual length of the fibers within the system, because each fiber measurement included in the average is weighted equally. Conversely, L_w_ is particularly valuable in representing the effect that longer fibers have on the distribution and are weighted to account for the occurrence of longer lengths. It would be expected that longer fibers are able to achieve higher levels of load transfer and, therefore, L_w_ may be more relevant to the resulting mechanical properties.

**Figure 2:**
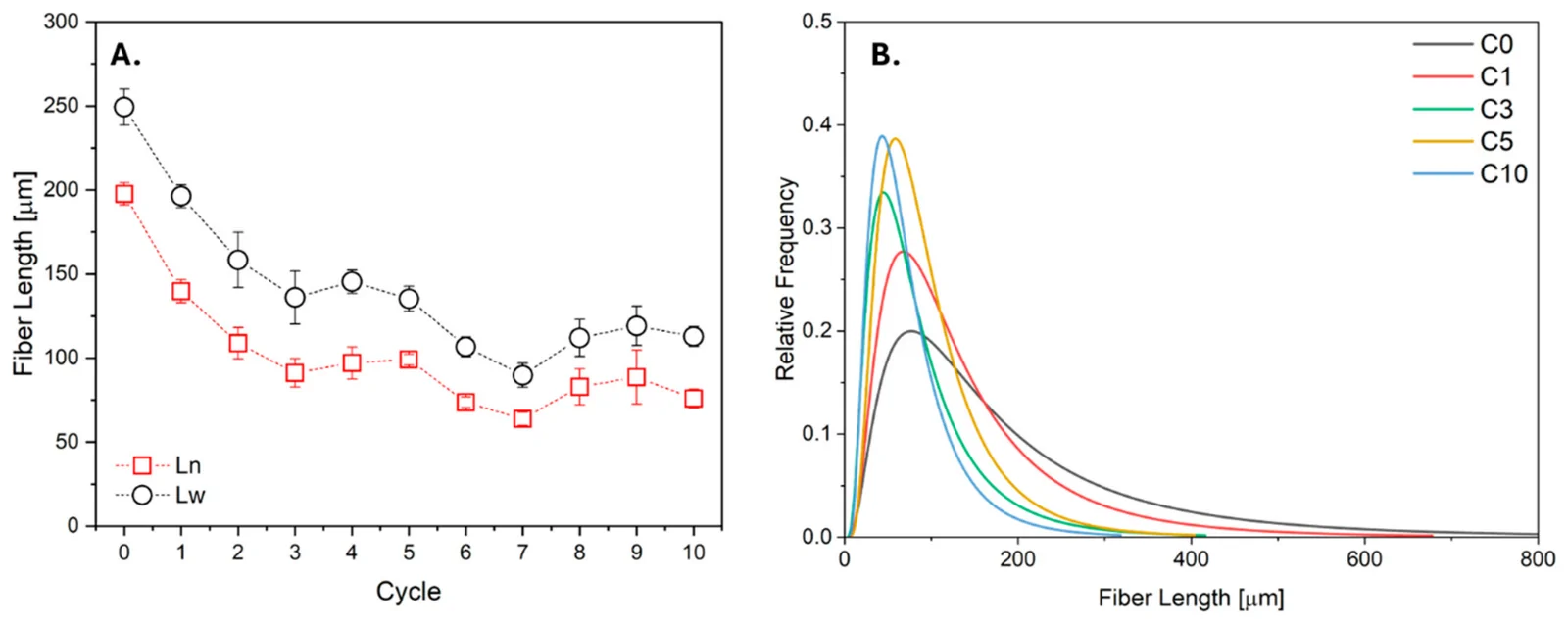
(**A**) The weight and number average fiber lengths of each cycle with standard deviation, measured from pellets after re-extrusion; (**B**) log-normal distributions of the measured fiber lengths which illustrate the increased frequency of fibers less than 100 micrometers with subsequent re-extrusion.

When assessing the L_n_ (Figure 2A), it was found that the most severe fiber breakage occurs during the first re-extrusion cycle (C0 → C1), where L_n_ decreased 29%. Furthermore, it was found that the rate of breakage slows with each additional extrusion cycle, showing stabilization behavior between C3–C5 and exhibiting asymptotic behavior between C6–C10. L_n_ asymptotically approached a terminal average length of ~75 µm, representing a 55% reduction in L_n_ after 10 TSE re-extrusion cycles. This reduction can also be seen in the sharpening of the log-normal distribution curves of the length measurements (Figure 2B), highlighting the increased frequency of short fibers after each extrusion cycle. L_n_ also notably exhibited exponential decay behavior. The small increases in average fiber length observed between C3–C4 and C7–C8 are attributed to sampling/measurement variability arising from unequal fiber counts per cycle, rather than a true reversal of fiber attrition.

The decay behavior is of significance because it demonstrates that fiber length is conserved over multiple re-extrusion cycles [25]. As such, the corresponding result of fiber breakage is an increase in the number of short fibers within the system, fundamentally changing the morphology of the composite with every additional re-extrusion process until a terminal length is achieved; the increasing number of fibers within the system should exhibit inverse exponential growth behavior when compared to fiber length. The formation of composite microstructure (e.g., fiber orientation and/or distribution) in short fiber composite materials not only depends on the fiber length but also the number of fibers within the material system, as well as how they interact and orient themselves, which can lead to changes in material performance [40,44].

Degradation of fiber length can also be particularly important for thermomechanical properties such as storage modulus, loss modulus, and damping factor that are sensitive to filler shape and composite morphology [45,46]. It must be noted that the value of the terminal fiber length identified is dependent not only on the initial fiber length within the material but also the matrix viscosity, the intensity of the shear stress, and the specific processing methods and conditions [28].

Similar to L_n_, L_w_ also exhibited exponential decay behavior, with the most severe reduction occurring after the first re-extrusion cycle (21%) and average stabilization and asymptotic behaviors between C3–C5 and C6–C10, respectively. When comparing C0 to C10, the L_w_ decreased 62%. L_w_ is valuable for representing the effect of length on resulting mechanical properties because it can more appropriately account for the greater load-bearing contributions provided by longer fibers within the system. The prediction of tensile strength and a more detailed analysis of the analytical trends of L_w_, as they relate to mechanical properties, will be presented in a later section. Considering that there is minimal evidence of matrix degradation and no significant loss of fiber volume, it is expected that resulting material behaviors and mechanical performance will be dependent on the degradation of fiber length and are expected to exhibit exponential decay behavior at a similar rate to that of the fiber length.

### 3.3. Processing Behavior

Viscoelastic behavior observed from rheological characterization is a valuable tool to gain insight into the structure of a material and how it responds to deformation. Viscoelasticity is especially important for complex materials like fiber-filled polymers such as CF-ABS as their processing characteristics and microstructural formation are highly dependent on the aspect ratio of the fiber. In addition, rheological properties such as complex viscosity are sensitive to both molecular weight and fiber length [28,47,48], where in a previous study by this team [10] it was found that fiber length degradation was the dominant degradation mechanism of rheological properties in recycled CF-ABS [10]. As GPC and FTIR showed minimal matrix degradation, the impacts of fiber length reduction on material viscosity after TSE re-extrusion were evaluated via small-amplitude oscillatory frequency sweeps. The materials were tested at 235 °C, a slight increase from the maximum barrel temperature in the TSE process, to account for shear heating potential in the process [49] that results in a hotter melt at the die. Previous research has shown that melt temperatures during extrusion are typically higher than the barrel temperature due to viscous heating, a phenomenon that occurs due to the frictional forces within the TSE system [50]. To quantify the effect of fiber length degradation on the observed complex viscosity, comparison values were selected from low (10 rad/s) and high (628 rad/s) angular frequency regions for each cycle to compare to pristine CF-ABS (C0). These comparison values were chosen as previous work [10,48] has identified that the effect of fiber length on the resulting material viscosity is more prominent at low shear rate regions compared to high shear rate regions.

**Figure 3:**
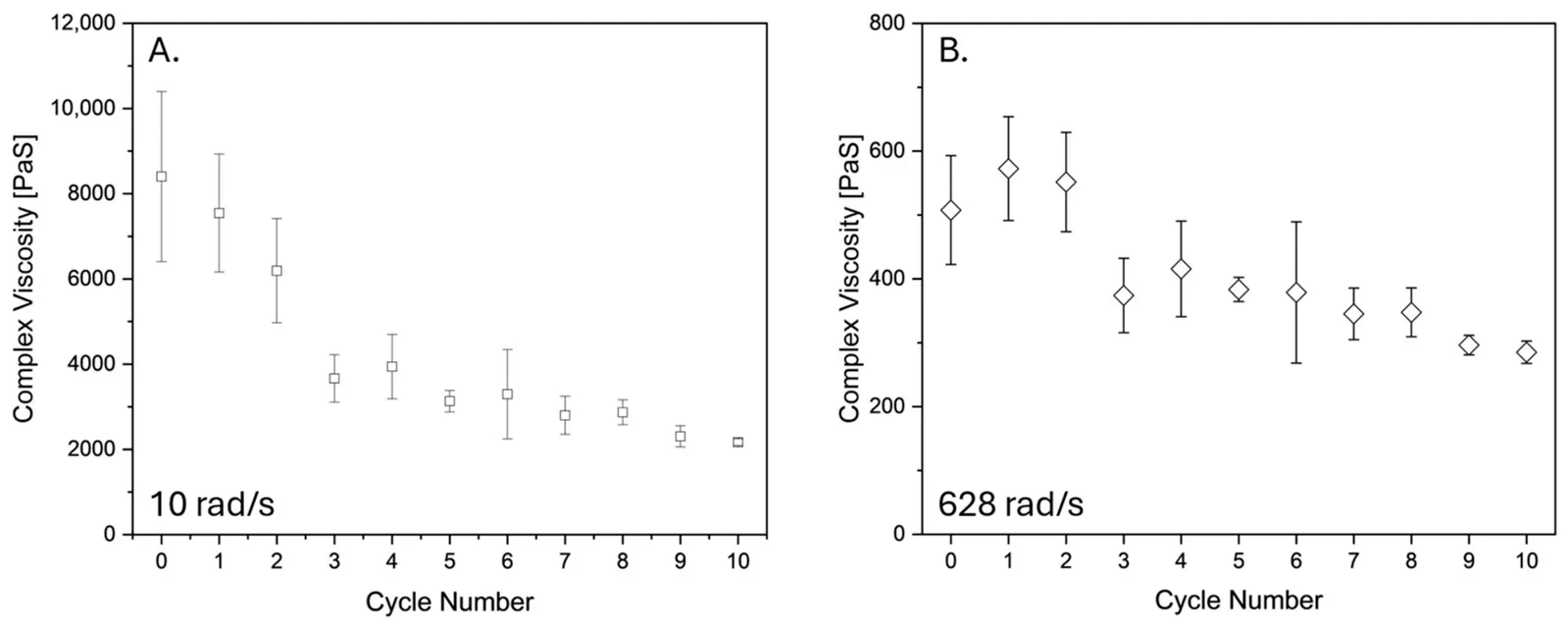
Average complex viscosity measurements and standard deviations of C0–C10 at (**A**) 10 rad/s, where there is the most pronounced separation in complex viscosity and (**B**) at 628 rad/s where there is significant but less severe separation in complex viscosity. The complete complex viscosity curves are illustrated in Figure S4.

At low angular frequency (Figure 3A), a decrease in complex viscosity during the first three re-extrusion cycles (C1–C3) was observed. It was found that there was a 10%, 26%, and 56% decrease in complex viscosity of C1, C2, and C3, respectively when compared to C0. The severe drop in viscosity at C3 could be indicative of reduced effects of rigid percolation of the CF [51,52,53], which is typically seen at the onset of non-linear behavior. Rigid percolation effects require further investigation with respect to this dataset. After C3, stabilization of the complex viscosity was observed with respect to the number of re-extrusion cycles, where the rate of decrease in the complex viscosity slows between C3–C5 and is relatively constant between C6–C10. After 10 re-extrusion cycles, the CF-ABS material exhibited a 74% decrease in complex viscosity when compared to C0. This stabilization behavior is echoed when observing the complex viscosity at 628 rad/s (Figure 3B), where it was found that for C0–C2 there were minimal differences in complex viscosity, though trending downward and the complex viscosity of C10 was 44% lower than that of C0. As discussed briefly above, fiber length plays an important role in the viscosity of a fiber-filled material, as rheological behavior can be used to better understand the shear rates experienced during manufacturing [54,55], especially for extrusion and injection molding processes. For example, extrusion or injection molding of fiber-filled materials results in parts with complex microstructures, where fibers are most likely to be preferentially oriented in the direction of the material flow due to shear, likely influencing the resulting mechanical properties [56]. The comparative results from both low and high frequency regions show that the material asymptotically approaches terminal viscosity behavior which is highly dependent on the fiber length as a function of re-extrusion.

### 3.4. Thermomechanical Properties

**Figure 4:**
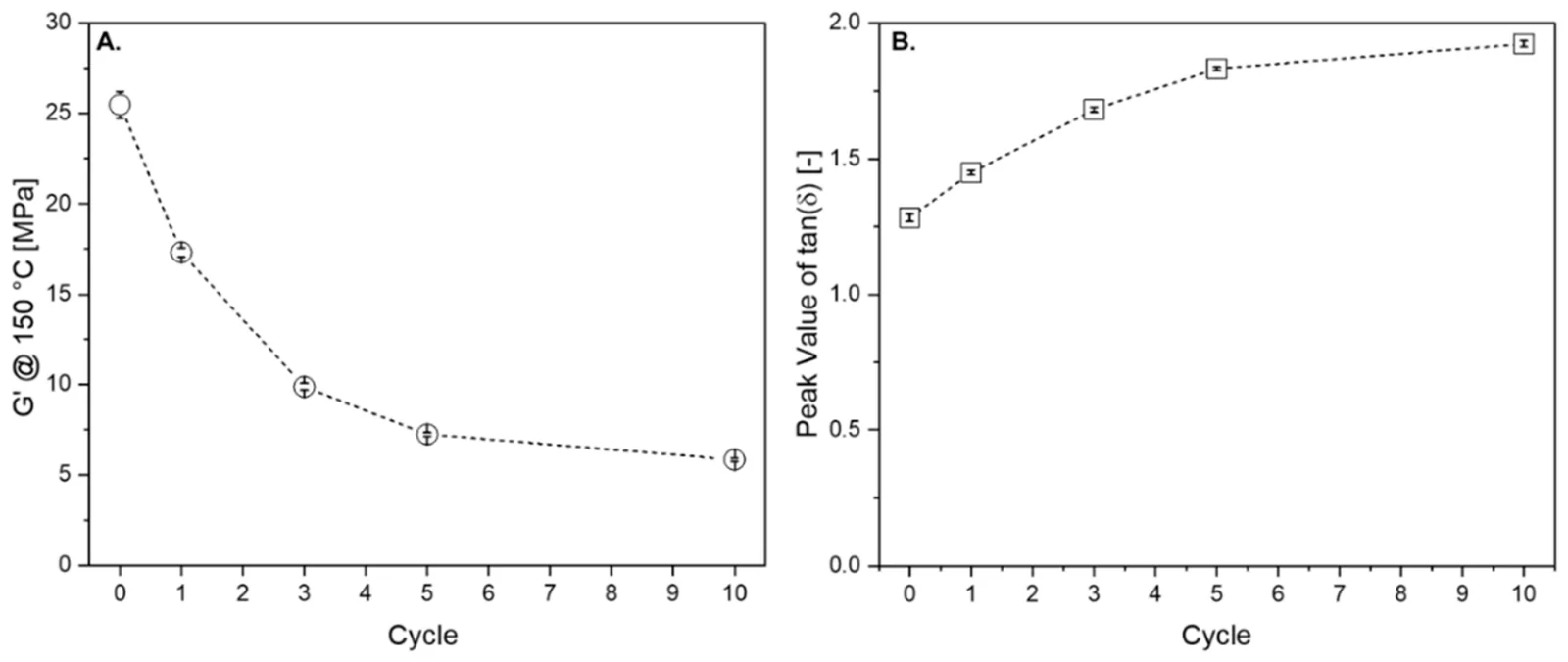
Dynamic mechanical analysis (DMA) results showing changes in (**A**) storage modulus (G′) and (**B**) damping behavior (tan (δ)) with respect to the number of thermomechanical reprocessing cycles. Results illustrate a non-linear reduction in stiffness and a non-linear increase in damping behavior with subsequent reprocessing. Complete G′. G″, and tan(δ) curves are reported in Figure S5. The error bars in (**A**,**B**) illustrate the standard deviation of the triplicate.

To further elucidate the influence of fiber length degradation on the resulting properties of CF-ABS, DMA was performed on injection-molded samples. DMA is another valuable tool for understanding and characterizing the structural behavior of fiber-filled materials as it allows for observation of the elastic and viscous response of a material with respect to time, temperature, or frequency. Properties such as storage modulus (G′), loss modulus (G″), and the damping ratio (tan(δ)) are influenced by both matrix phase and fiber phase contributions such as (1) the morphology of the matrix material, (2) the size, shape, and concentration of filler, and (3) the strength of the interfacial bond between the two phases. This is especially of interest for recycled fiber-filled materials that have experienced significant fiber breakage. Due to fiber breakage, there is a corresponding increase in the number of short fibers within the system (cf. Figure 2B) and, therefore, an increased surface area to volume ratio of available sites for interactions between the matrix and the fiber. It was expected that DMA would allow for the observation of the change in dynamic properties as a function of the fiber breakage that occurs with re-extrusion. Selective cycles of C0, C1, C3, C5, and C10 underwent a temperature ramp to gain insight into potential changes in structural characteristics due to the re-extrusion process. The storage modulus of a material represents the elastic, or solid-like, portion of viscoelasticity and is often used to describe the stiffness of a material under oscillatory loads. To isolate the effects of fiber breakage on storage modulus, G′ was observed at 50 °C (Figure S6) and 150 °C (Figure 4).

At 50 °C, the storage modulus can aid in providing insight into the stiffness of the material as a solid. It was found that there was a significant (*p* < 0.05) reduction in the storage modulus at 50 °C when comparing C0 to C10 (cf. Figure S6); however, the G′ of C10 only exhibits a 9.5% decrease when compared to C0. In contrast, when the G′ is observed at 150 °C (T_g_ + 50 °C) there are significant reductions in G′ which also exhibit exponential decay behavior. When comparing C0 to C10 at 150 °C, a 77% reduction in storage modulus is observed. This is important because CFRP materials like CF-ABS are used most often in extrusion and injection applications, where stiffness while cooling during manufacturing is critical for behaviors like shrinkage and warping.

It can be seen in Figure 4A that the storage modulus exhibits exponential decay with respect to the number of re-extrusion cycles, similar to that of the fiber length degradation, and is most pronounced at elevated temperatures (150 °C); this indicates that the material is less stiff and shows lower resistance to deformation after re-extrusion. This behavior in unfilled materials is typically related to the degree of entanglement of the polymer itself, but for short fiber composite materials we can postulate that this behavior describes the entanglement between the fibers and the matrix. In previous research [57,58], it has been suggested that increased G′ and G″ behavior in short fiber composite materials is due to both the load-bearing capacity of the fiber, which increases with length, and slowed polymer chain relaxation dynamics due to spatial hindrances caused by the presence of fibers.

Further, the damping capacity, as described by the tan(δ) of a material, has also been used in recent publications to describe the interfacial region between fiber and matrix materials in composites [45,59], particularly for materials that utilize sizing to improve composite performance. Tan(δ) also describes the ratio of elastic response to viscous response and can provide insight into viscoelastic regime shifts. It was observed (Figure 4B) that the maximum peak value of tan(δ) increased with respect to fiber breakage until a stable fiber length was achieved between C3–C5. Asymptotic behavior was observed between C6–C10, where the maximum value of tan(δ) was found to be 1.96. The tan(δ) of neat ABS has been documented previously to be approximately 2.1 [60]. These results indicate that with respect to fiber breakage, the material becomes softer and more dominated by resin behavior in response to applied deformation, suggesting that with increasing re-extrusion cycles, the viscoelastic properties of the re-extruded CF-ABS become more similar to the matrix behavior alone as the fiber lengths become shorter and more uniform (cf. Figure 2B). These results could be indicative of changes in the interfacial bonding behavior and/or diminished rigid percolation behavior, like what is seen in particle-filled material systems. The tan(δ) results follow an inverse exponential growth relationship to the trends observed with average fiber length and a parallel trend to the exponential growth rate of the number of particles within the system (cf. Figure 2B). Previous literature has also shown that as fiber length is reduced, the viscoelastic properties of short fiber composite materials may approach that of their neat counterpart [61], results that are in close alignment with the observed behavior in this study.

### 3.5. Mechanical Properties

The fiber length of any fiber-filled composite is critical to the load transfer process. Typically in discontinuous fiber-reinforced composites, the goal is to achieve a fiber length that is greater than or equal to the critical fiber length for the material system, which is ~375 μm when assuming a fiber strength of 4000 MPa [11]. However, many short fiber discontinuous materials used for extrusion or injection processes have been found to have average fiber lengths below the critical fiber length [8,10,62] (Figure 2A, C0), suggesting that as a pristine material they are unable to achieve peak load transfer and therefore peak mechanical properties.

**Figure 5:**
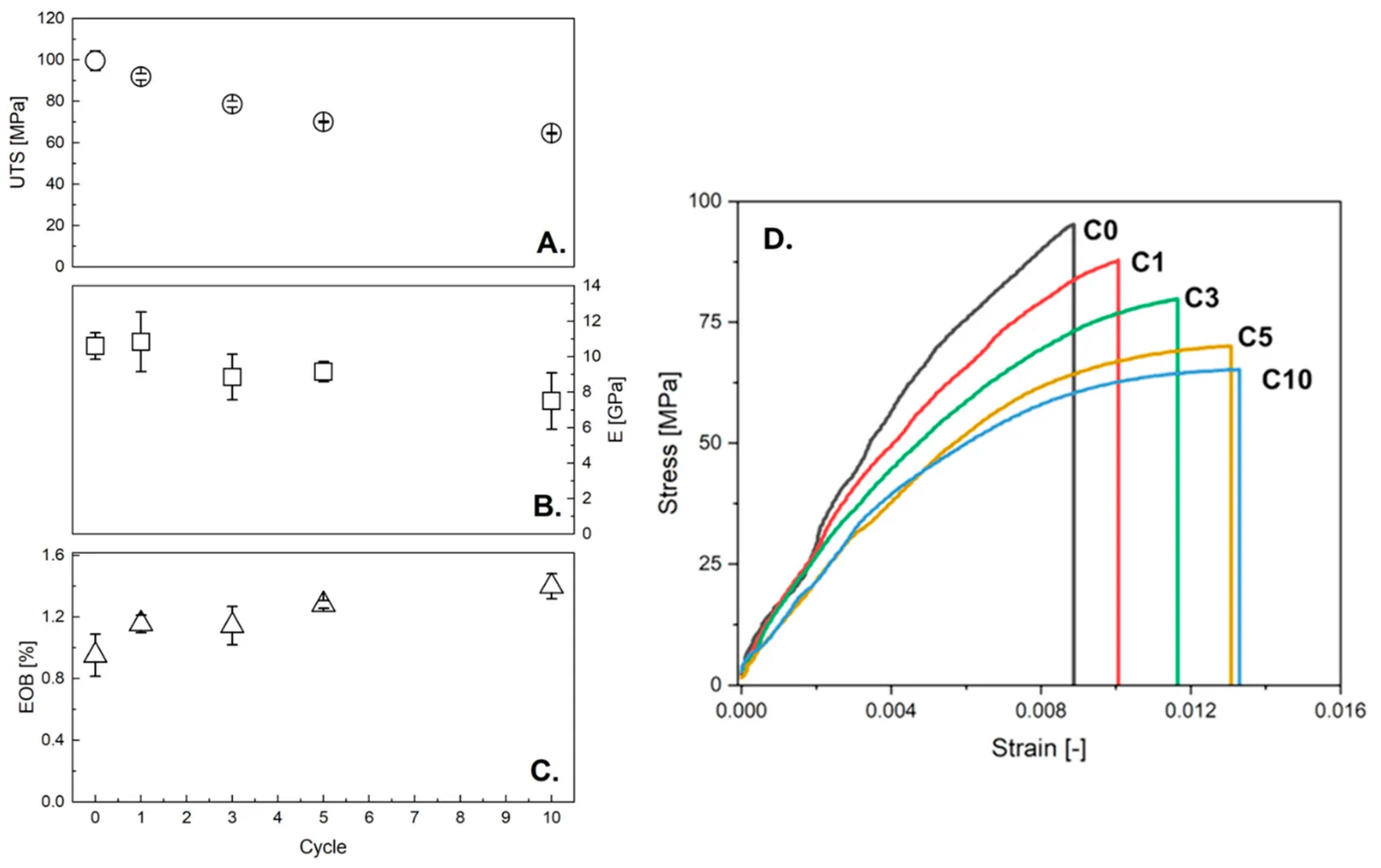
The resulting mechanical properties (**A**) UTS, (**B**) Young’s modulus, and (**C**) elongation at break. (**D**) The representative stress–strain curves of tested tensile specimen. These curves illustrate how the increasing EB, with respect to cycle, aids in maintaining the fracture toughness of the material, even at overall lower peak loads. The fracture toughness of the material was calculated as the area under the curve of the stress–strain curve produced from tensile testing. The average values and standard deviations for each property are reported in Table S5.

The mechanical properties of C0, C1, C3, C5, and C10 were evaluated by injecting molding specimens and observing the UTS, elastic modulus, fracture toughness, and elongation at break (EB) (Figure 5 and Table S5). Injection molding was utilized in this study to not only demonstrate the manufacturability of CF-ABS but also to limit microstructural defects and artifacts that may be more difficult to control in extrusion-based processes like LFAM. There was a significant (35%) reduction in UTS after 10 re-extrusion cycles when comparing C0 and C10. The tensile strength behavior with respect to cycle (Figure 5 and Table S5) was also observed to exhibit the same exponential decay behavior identified in the fiber length degradation, the rheological behavior, and the dynamic mechanical behavior with respect to cycle.

It is reasonable to conclude that the dominant degradation mechanism of the UTS was fiber length degradation. It was also found that elastic modulus decreased 29% when comparing the average value of C10 to C0. The decrease in Young’s modulus is statistically significant (*p* < 0.05) although overlapping standard deviations indicate there was sample variability.

In Figure 5 the re-extruded samples achieve lower peak loads compared to the pristine material but are also able to maintain those loads over larger strain values, resulting in a 46% increase in EB when comparing C0 to C10. EB behavior shows a similar exponential growth behavior similar to the tan(δ) behavior seen in Figure 4B, indicating that as the fiber length degrades, the material approaches the EB of neat ABS. The EB for comparable grades of neat ABS is reported to be 1.8% [63]. It should be noted that Boldizar et al. [22] also identified an increase in EB after re-extrusion. However, it is postulated that the mechanism for the EB increase is a function of the degradation of fiber length rather than a chemical change in the ABS matrix, representing a transition from fiber-dominant mechanical behavior to matrix-dominant mechanical behavior. As a result of the increased ductile behavior, it was found that there was no significant difference in the material’s fracture toughness.

### 3.6. Non-Linear Curve Analysis and Prediction of Tensile Strength

As the UTS data adheres closely to the trends of fiber breakage with respect to cycle, it is important to consider if this property degradation can be modeled and predicted for re-extruded materials. For short discontinuous fiber-filled materials, the Kelly–Tyson (KT) model is one of the most common methods to predict tensile strength [44,64]. The KT model is a modified rule of mixtures model that when coupled with fiber length, the interfacial shear strength between the matrix and fiber, and the orientation efficiency of the fiber has been able to reasonably predict the resulting strength of discontinuous fiber-filled composites. However, when considering composite recycling, attributes such as the interfacial shear strength and the orientation efficiency may change with a reduction in fiber length that occurs with each cycle. As such, the values for interfacial shear strength are estimated as $\sigma_{m}\;/\sqrt{3}\;$, where $\sigma_{m}$ is the UTS of the neat matrix (64.5 MPa) [65,66], and the orientation efficiency can be calculated using an assumed initial strength value (Table S6). However, without directly observing changes in these variables with each re-extrusion cycle, they must be assumed constant; this assumption leads to potential discrepancies in predictions. Therefore, it is proposed that reducing the number of required inputs, utilizing the exponential decay rate of fiber breakage, and an exponential decay analytical model, may aid in better predictions of resulting tensile strength after re-extrusion.

Based on the results of the degradation analysis, it was postulated that the fiber length degradation could be used as a causal variable to better predict the resulting mechanical behavior. The weighted-average fiber length was determined to be the dominant degradation indicator and the fiber length vs. cycle trends (cf. Figure 2A, L_w_) were curve fit in JMP using a three-parameter exponential decay equation. This is consistent with approaches taken previously in the literature [28]. The average decay rate (*k*) for the weighted-average fiber length was found to be 0.47 per cycle, with a 95% confidence interval (CI) ranging from 0.67 (lower 95th CI) to 0.27 (upper 95th CI). These values were found to be statistically significant (*p* < 0.0001) and the R^2^ value was 94%.

To predict the resulting UTS values with respect to cycle, a smooth fiber length curve for L_w_ was produced using Equations (1)–(3), where *a* and *b* are derived from the curve fitting process and represent the terminal fiber length (L_T_) subtracted from the initial fiber length (L_0_) and the terminal fiber length (L_T_), respectively.

(1) $$\bar{L\left(N\right)}=\mathrm{aexp}\left(-\mathrm{kN}\right)+b$$

(2) $$a=L_{0}-L_{T}$$

(3) $$b=L_{T}$$

(4) $$a+b=L_{0}$$

In order to adapt the exponential decay model for the prediction of UTS, the decay equation was adapted with the following changes to account for strength contributions from the pristine composite (σ_c,i_) and the neat matrix material (σ_m_):

(5) $$\bar{\sigma \left(N\right)}=\mathrm{aexp}\left(-\mathrm{kN}\right)+b$$

(6) $$a=\sigma_{c,\;i}-\sigma_{m}$$

(7) $$b=\sigma_{m}$$

(8) $$a+b={\;\sigma }_{c,i\;}$$

UTS is expected to follow the same exponential decay behavior as fiber length, suggesting that the fiber length decay rate can be used as a representative value for UTS degradation with repeated re-extrusion (Equations (5)–(8)). Equations (5)–(8) only require three unique inputs: the decay rate of fiber length, the UTS of the pristine material, and the UTS of the neat matrix. The KT model, on the other hand, requires eight inputs. While many of these values are easy to find and/or calculate, reducing the number of variables needed to be assumed in predictions can be beneficial when properties like interfacial shear strength and orientation efficiency may be difficult to observe, find consistently in the literature, or may change with fiber length degradation.

**Figure 6:**
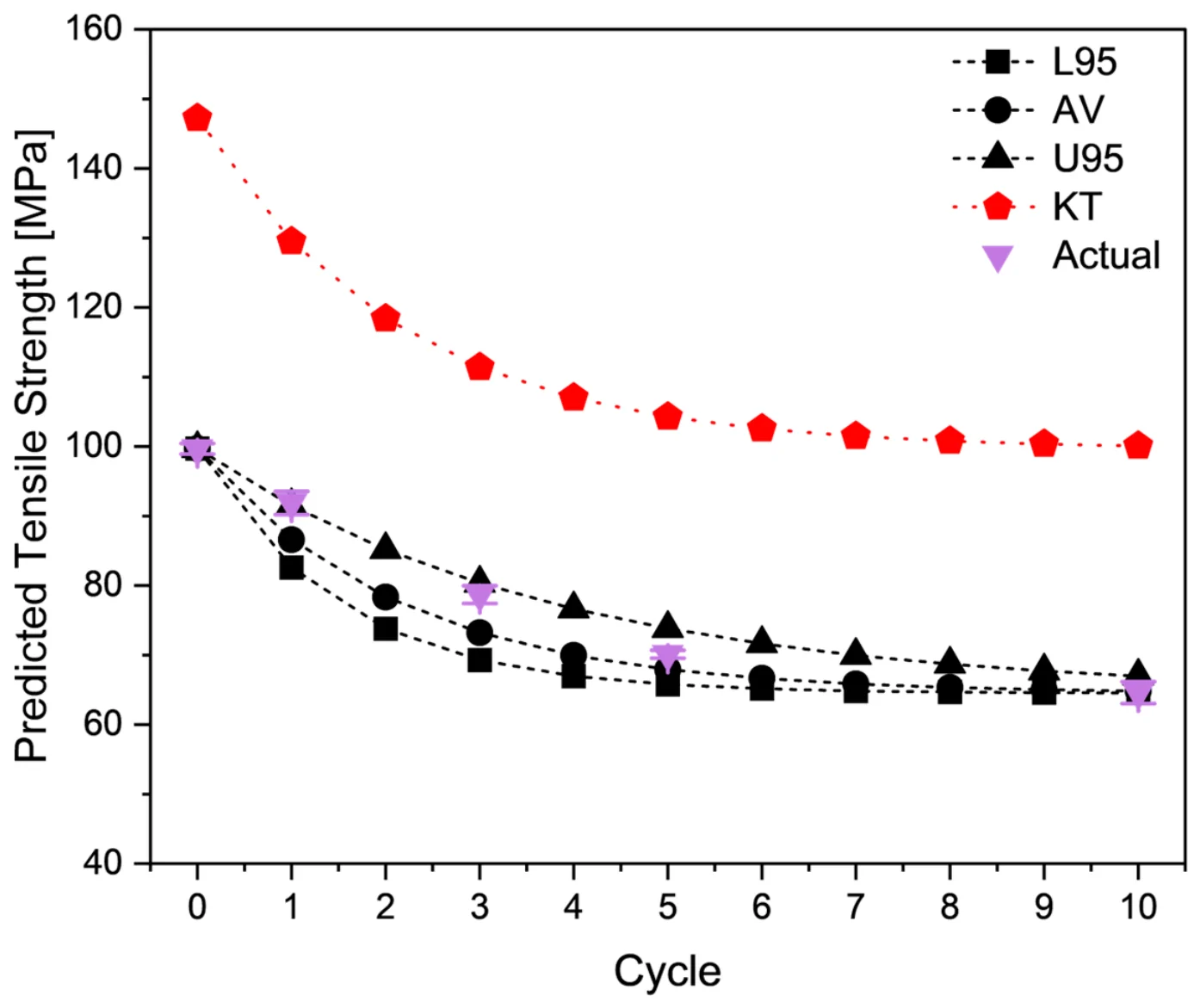
UTS predictions; KT model predictions (red) and the exponential decay model (black) where the initial strength is equal to the observed UTS for C0 (cf. Table S5). To illustrate the sensitivity to initial strength values, model curves are illustrated in Figure S6.

Using the decay rate values from the confidence interval for L_w_, an initial composite strength of 99.7 MPa (cf. Table S6), and a neat resin strength of 64.5 MPa [67], three resulting predictor formulas were calculated. For completeness and comparison, the KT model was also used to predict the UTS as a function of cycle number (Figure 6). For the KT model, fiber length was described by the smooth fiber length curve produced by Equations (1)–(4) with a decay rate of 0.47 per cycle.

Figure 6 illustrates the results of the exponential decay equations (black) and the KT model (red) using the input parameters detailed above. The KT model overpredicts the tensile strength values for all cycles and terminates at a higher strength than those predicted with the use of the analytical trends. It is postulated that the difference in the values is due to the potential for over-estimation because of the use of a constant orientation efficiency value (0.55 [-]) and a constant value for the interfacial shear strength (37.2 MPa). While not directly measured, it can be inferred from the resulting values of tan(δ) with respect to cycle that increasing values of tan(δ) can correspond to a decrease in interfacial adhesion [68], supporting the proposal that interfacial shear strength may decrease. A change in interfacial strength can also be inferred by the total number of fiber ends exhibiting exponential growth with respect to cycle number until a terminal length is achieved. It should also be noted that the exponential decay model can overpredict C0 (Figure S7A), as this model is very sensitive to the initial composite strength value assumption (Figure S7B).

However, the exponential decay model predicts UTS values that agree more closely with the experimentally observed UTS. For the first three cycles, the measured UTS aligns more closely with the decay rate defined by the upper 95th percentile of the confidence interval and then trends towards the average decay rate prediction in cycles 5–10. Overall, the model exhibits slight underprediction but achieves improved predictive performance while requiring fewer inputs. The root mean squared error (RMSE) of the residuals (Experimental minus Predicted) is significantly reduced from ~38 (KT model) to ~3.5 when comparing the values reported for the average decay rate equation. Further, even when the model uses an initial value reported from a technical data sheet (110 MPa), rather than the observed UTS, the RMSE remains low (~4.8).

A potential limitation of this model is that it requires fiber length characterization; this can be difficult for some recycling streams and remanufacturing pathways where the previously experienced thermomechanical degradation may not be known or easily quantified. However, this model represents early efforts to better understand how recycled material performance can be predicted. Further, due to the statistically significant correlation between fiber length and tensile strength, it was important to compare the decay rate of fiber length to the decay (or growth) rates of the resulting material behaviors evaluated in this study. Table 1 highlights the degradation indicators, or causal variables, identified in this study as having the potential to cause a reduction in performance; it can be seen that fiber breakage is the primary degradation mechanism for CF-ABS. To better understand the correlation between fiber length and the resulting properties, the rates of decay, or growth in the case of tan(δ), were curve-fit using a three-parameter exponential decay/growth equation. The upper and lower 95% confidence interval for the k values of each property, while not duplicative in comparison to the decay rate of L_w_ do show statistically significant correlation.

The confidence intervals reported for each resulting property are reasonably captured by the confidence interval for the decay rate of L_w_, which shows the significant influence fiber breakage has on the performance and properties of re-extruded CF-ABS. Therefore, it may be possible to model and predict these properties as well, which is significant because property predictions can be difficult to perform over multiple lifespans of recycling and remanufacturing. In applications and industries that may require specific mechanical properties or material performance, being able to reasonably and simply predict that performance can aid in determining the maximum lifespan of a material before it needs to be downcycled or disposed of.

## 4. Conclusions

After 10 re-extrusion cycles, it was found that there was minimal evidence of chemical degradation of the ABS matrix, highlighting the robustness of the ABS matrix. It was found that the primary degradation mechanism was fiber breakage, confirming fiber length reduction as the dominant driver governing the change in material behavior and performance with repetitive re-extrusion. Fiber breakage exhibited exponential decay behavior that began stabilizing in cycles C3–C5, followed by asymptotic behavior for both L_n_ and L_w_ in cycles C6–C10; L_n_ and L_w_ decreased 55% and 62%, respectively.

Complex viscosity exhibited exponential decay behavior that was most pronounced in low-shear rate regions; complex viscosity decreased 74% at low-shear rates and 44% at high shear rates. Mechanical performance was assessed by DMA and tensile testing. G′ decreased exponentially with cycle number, with the trend most pronounced at elevated temperatures; decreasing by 77% compared to C0. Notably, tan(δ) exhibited an inverse growth trend when compared to fiber length, indicating that the material becomes softer and increasingly behaves more like the neat ABS resin after fiber breakage; peak tan(δ) values approach the peak values for unfilled, neat ABS. This behavior, together with the observed increase in elongation at break (46%), reinforces the conclusion that the material transitions from fiber-dominant to matrix-dominant mechanical behavior as fibers are broken and their lengths become more uniform.

The UTS data also showed exponential decay, with the most severe reduction occurring between C0–C1; after 10 re-extrusion cycles, the UTS decreased by 35% compared to C0. As the UTS data trends closely aligned with those identified in the fiber length assessment, a simple analytical model was developed. It was found that the exponential decay rate of L_w_ could be used to describe the exponential decay rate for predicting UTS, and the model presented in this study showed a significant reduction in RMSE values compared to the KT model for discontinuous short fiber composites. It can be concluded that the rate of decay or growth of the material performance evaluated in this study is statistically correlated to the rate of fiber length (L_w_) degradation. Overall, these results highlight the significant impact fiber breakage has on resulting processing behavior and mechanical properties, while demonstrating that thorough fiber length characterization can enable improved prediction of resulting behaviors and performance before manufacturing. Future work should focus on extending these models to other fiber-filled thermoplastic materials that are of interest for recycling.

## Figures and Tables

**Table 1:** Trends in M_w_, L_w_, η*, G′, tan(δ), and UTS as a function of the number of TSE reprocessing cycles. Both M_w_ and L_w_ were evaluated as potential causal variables for the observed property changes. Because M_w_ showed no significant change across cycles, it was determined that the trends are correlated the most with L_w_.

Variable		L95	Avg.	U95
***Potential Causal Variables***	M_w_	-	-	-
	L_w_	0.67	0.47	0.27
***Response Behavior***	Complex Viscosity (10 rad/s)	0.45	0.31	0.17
	G′ @ 150 °C	0.54	0.53	0.52
	Peak Tan(δ)	0.36	0.31	0.25
	UTS	0.35	0.28	0.21

## Data Availability

The original contributions presented in this study are included in the article/Supplementary Materials. Further inquiries can be directed to the corresponding author.

## Supplementary Materials

The following supporting information can be downloaded at: https://www.mdpi.com/article/10.3390/polym18212634/s1. Table S1: Twin screw extrusion temperature profile; The temperature of the melt at the die was 234 °C; Table S2: Injection molding parameters; Table S3: Tg values are reported for all tested samples via DSC and DMA. Triplicates were performed for each measurement. There was no significant reduction in Tg, indicating that the polymer phase of the material has not undergone substantial material degradation, as confirmed by the Mw measurements via GPC and the spectra observed from FTIR; Table S4: TGA was utilized to assess the thermal stability of all material samples and to determine the residual fiber content present in the materials at 600 °C. There were minimal change in thermal stability when comparing C0 to the re-extruded cycles. There was no significant change in residual fiber weight percentage when comparing C0 to the re-extruded cycles; Table S5: Mechanical properties observed of select samples (C0, C1, C3, C5, C10) of re-extruded CF-ABS; Table S6: The following parameters were utilized in the KT-model predictions; Figure S1: Average extruder torque (%) observations with standard deviation. The extruder torque showed a higher value in the processing of C1, which is where the most significant fiber breakage occurred. Torque values were relatively constant across C2–C10; Figure S2: Weight-average molecular weight (A), number-average molecular weight (B), and dispersity (C) values reported for all tested samples. Four samples were measured for each cycle, and the data are reported as the average and standard deviation associated with those replicate measurements. The results indicate that there is not a substantial change in molecular weight after 10 TSE re-extrusion cycles; Figure S3: A TA Instruments Discovery DSC was used to collect thermal properties on the re-extruded and pristine (C0) CF-ABS materials. The samples were tested with a heat–cool–heat ramp from 0 °C to 190 °C at 10 °C/min in an air environment. The glass transition temperature (Tg) was calculated using the inflection point of the second heat ramp. Triplicates were run for each cycle; Figure S4: Thermal stability data with respect to cycle number (data listed in Table S3); it can be seen that there is minimal change in thermal stability with respect to re-extrusion, trends over subsequent cycles show a relatively flat and constant trend; Figure S5: Frequency sweeps were utilized to observe the complex viscosity of all materials after every re-extrusion cycle. Assessing data from all cycles allows for a better understanding of the influence of degradation on resulting rheological behavior; Figure S6: Storage and loss modulus curves measured from DMA of select samples (C0, C1, C3, C5, C10). Triplicates were measured for each cycle; Figure S7: UTS predictions from the exponential decay model (black) where (A) illustrates model performance when UTSC is equal to 110 MPa, assumed from available data in literature and (B) illustrates model performance when UTSC is equal to the experimentally observed UTS of C0, 99.7 MPa. These results highlight the model’s sensitivity to σ_c,i_.
