# Supplementary material for: Quantifying the Impact of Multiple Twin-Screw Extrusion Cycles on Discontinuous Carbon Fiber Composites
Source: Polymers (Basel). 2026 Oct 23;18(21):2634. doi: 10.3390/polym18212634 (PMC13766140; doi:10.3390/polym18212634)
Supplement: Supplementary file 1 [file polymers-18-02634-s001.zip › polymers-4588409-supplementary.pdf]

Supplemental Information

Quantifying the Impact of Multiple Twin-Screw Extrusion Cycles on Discontinuous Carbon Fiber Composites

Roo Walker [1], Matthew Korey [2,*], Amber M. Hubbard [2], Daniel Webb [2], Evan J. Holt [3], Ally Collier [4], Katie Copenhaver [2], S. Michael Kilbey II [3], Soydan Ozcan [2] and Chad Duty [1,2,4]

[1] Bredesen Center for Interdisciplinary Research and Graduate Education, The University of Tennessee, Knoxville, TN 37996, USA; cduty@utk.edu (C.D.)
[2] Oak Ridge National Laboratory, Oak Ridge, TN 37831, USA
[3] Department of Chemistry, The University of Tennessee, Knoxville, TN 37996, USA
[4] Department of Mechanical and Aerospace Engineering, The University of Tennessee, Knoxville, TN 37996, USA
* Correspondence: koreym@ornl.gov

Keywords: carbon-fiber-reinforced thermoplastics; recyclability; pilot-scale manufacturing; fiber length; composite rheology

Table S1. Twin screw extrusion temperature profile; The temperature of the melt at the die was 234 °C.

**Zone**	**1**	**2**	**3**	**4**	**5**	**6**	**7**	**8**	**9**	**10**	**11**	**12**
**Temperature [°C]**	210	212	214	216	218	220	223	226	230	220	220	215

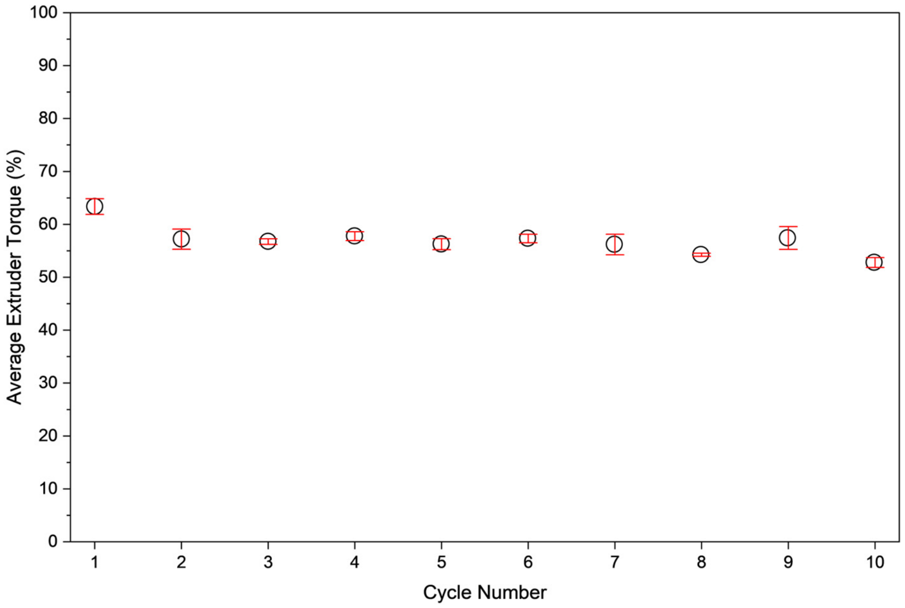

Figure S1. Average extruder torque (%) observations with standard deviation. The extruder torque showed a higher value in the processing of C1, which is where the most significant fiber breakage occurred. Torque values were relatively constant across C2-C10.

The following equations were used to determine the number-average and weight-average fiber lengths.

$$L_n = \frac{\sum_{i=1}^{n} l_i N_i}{\sum_{i=1}^{n} N_i} \quad (1)$$

$$L_w = \frac{\sum_{i=1}^{n} l_i^2 N_i}{\sum_{i=1}^{n} l_i N_i} \quad (2)$$

Table S2. Injection molding parameters.

**Zone**	**Zone Temperature (°C)**
1	235
2	245
3	250
4	255
5 – Injection	250

**Mold Temperature**	Room Temperature – No Active Heating
**Extrusion Pressure**	45 Barr

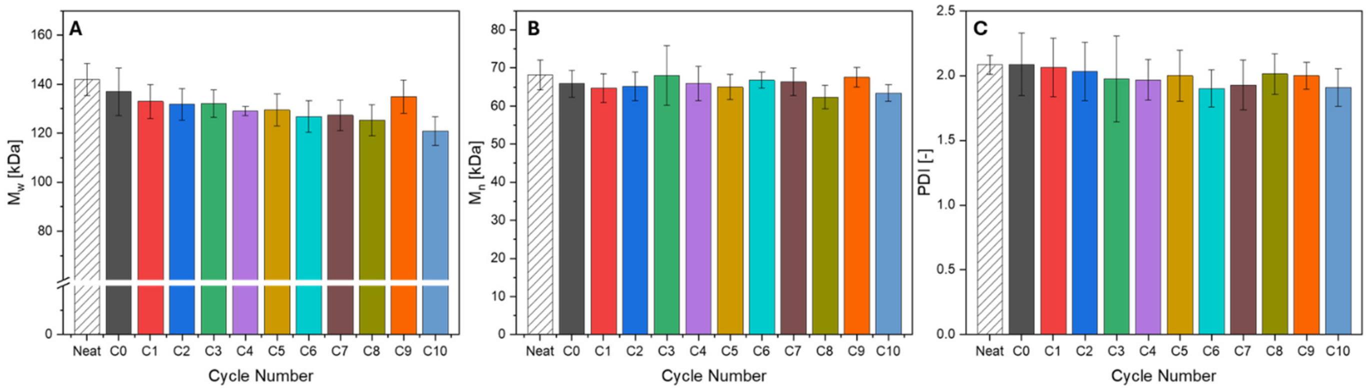

Figure S2. Weight-average molecular weight (A), number-average molecular weight (B), and dispersity (C) values reported for all tested samples. Four samples were measured for each cycle, and the data are reported as the average and standard deviation associated with those replicate measurements. The results indicate that there is not a substantial change in molecular weight after 10 TSE re-extrusion cycles.

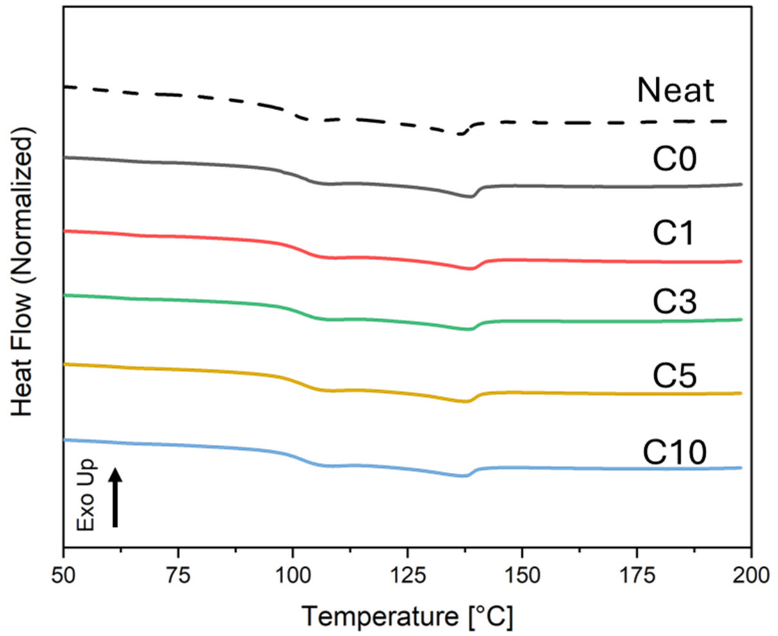

Figure S3. A TA Instruments Discovery DSC was used to collect thermal properties on the re-extruded and pristine (C0) CF-ABS materials. The samples were tested with a heat–cool–heat ramp from 0 °C to 190 °C at 10 °C/min in an air environment. The glass transition temperature (T_g) was calculated using the inflection point of the second heat ramp. Triplicates were run for each cycle.

Table S3. T_g values are reported for all tested samples via DSC and DMA. Triplicates were performed for each measurement. There was no significant reduction in T_g, indicating that the polymer phase of the material has not undergone substantial material degradation, as confirmed by the M_w measurements via GPC and the spectra observed from FTIR.

Material	DSC (Pellet)	E' Onset	Peak tan(δ)
**C0**	100.6 ± 0.2	108.4 ± 0.2	118.8 ± 0.5
**C1**	101.7 ± 0.4	107.6 ± 0.4	118.9 ± 0.3
**C3**	102.0 ± 0.5	108.2 ± 0.1	119.1 ± 0.1
**C5**	102.9 ± 0.5	108.5 ± 0.6	119.5 ± 0.5
**C10**	102.8 ± 0.1	108.2 ± 0.3	119.1 ± 0.5

Table S4. TGA was utilized to assess the thermal stability of all material samples and to determine the residual fiber content present in the materials at 600 °C. There were minimal change in thermal stability when comparing C0 to the re-extruded cycles. There was no significant change in residual fiber weight percentage when comparing C0 to the re-extruded cycles.

	$T_{2\%}$ (°C)	$T_{5\%}$ (°C)	T_{onset} (°C)	Wt% @ 600°C
**Environment**	*Air*	*Air*	*Air*	*UHP Argon*
Neat Pellet	309.1 ± 4.0	358.2 ± 2.0	387.9 ± 0.7	1.71 ± 0.03
C0	331.8 ± 2.0	365.5 ± 1.0	386.1 ± 0.8	21.04 ± 1.04
C1	336.1 ± 0.5	368.5 ± 0.4	396.0 ± 1.1	22.28 ± 0.04
C2	335.03 ± 2.0	368.3 ± 0.6	396.9 ± 0.2	22.20 ± 0.07
C3	335.3 ± 1.5	369.3 ± 1.0	395.1 ± 0.5	21.21 ± 0.07
C4	338.8 ± 4.2	370.9 ± 1.4	396.1 ± 2.8	21.22 ± 0.12
C5	341.9 ± 3.9	372.9 ± 1.1	395.3 ± 1.8	21.38 ± 0.15
C6	340.2 ± 1.9	372.0 ± 0.5	397.9 ± 1.6	21.43 ± 0.09
C7	339.6 ± 1.7	371.0 ± 0.8	396.5 ± 1.2	21.39 ± 0.07
C8	340.3 ± 1.9	370.9 ± 0.5	396.6 ± 0.4	21.50 ± 0.04
C9	337.7 ± 2.4	370.8 ± 1.3	396.2 ± 1.3	21.60 ± 0.2
C10	339.7 ± 1.1	372.1 ± 0.5	396.3 ± 1.0	21.56 ± 0.08

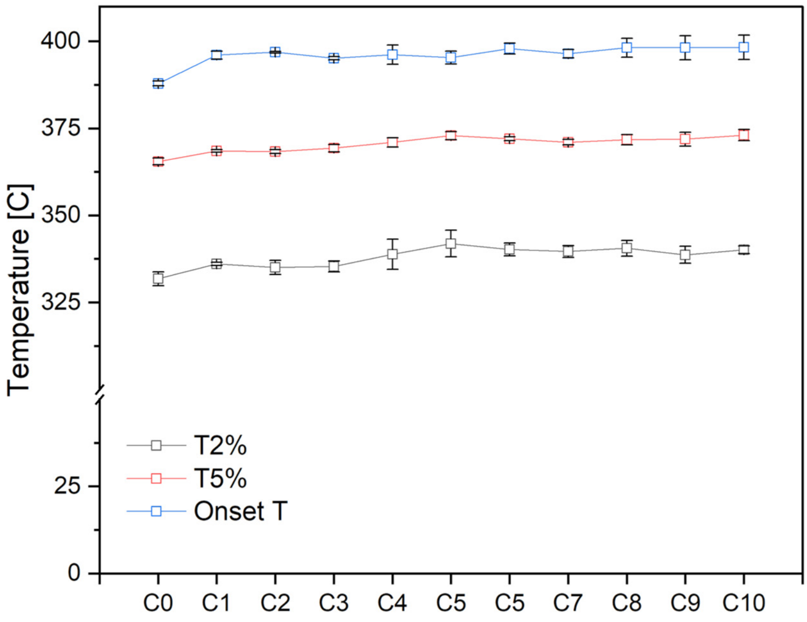

Figure S4. Thermal stability data with respect to cycle number (data listed in Table S3); it can be seen that there is minimal change in thermal stability with respect to re-extrusion, trends over subsequent cycles show a relatively flat and constant trend.

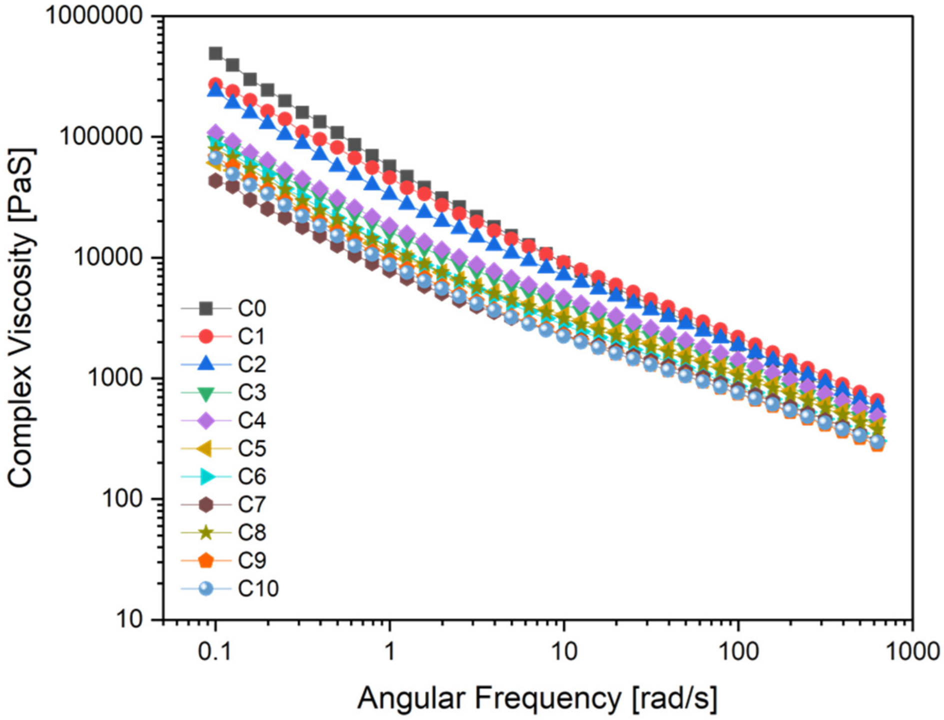

Figure S5. Frequency sweeps were utilized to observe the complex viscosity of all materials after every re-extrusion cycle. Assessing data from all cycles allows for a better understanding of the influence of degradation on resulting rheological behavior.

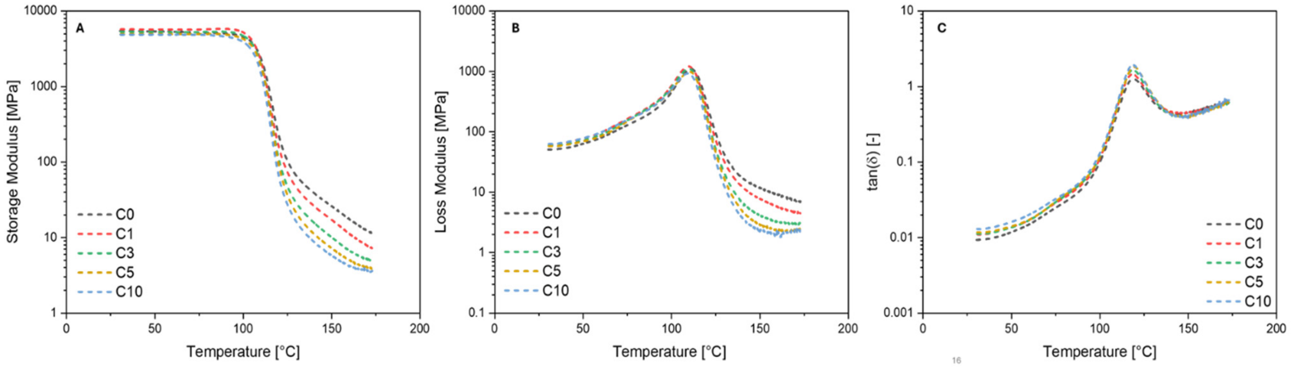

Figure S6. Storage and loss modulus curves measured from DMA of select samples (C0, C1, C3, C5, C10). Triplicates were measured for each cycle.

Table S5. Mechanical properties observed of select samples (C0, C1, C3, C5, C10) of re-extruded CF-ABS.

Cycle Number	UTS	Elastic Modulus	Fracture Toughness	Elongation at Break
	MPa	GPa	MPa	%
**C0**	99.7 ± 4.8	10.7 ± 0.8	0.60 ± 0.1	0.95 ± 0.14
**C1**	91.9 ± 1.6	10.8 ± 1.7	0.67 ± 0.03	1.15 ± 0.06
**C3**	78.7 ± 1.5	8.9 ± 1.3	0.58 ± 0.1	1.14 ± 0.12
**C5**	70.1 ± 0.4	9.2 ± 0.6	0.62 ± 0.01	1.28 ± 0.03
**C10**	64.6 ± 0.4	7.5 ± 1.6	0.66 ± 0.03	1.39 ± 0.08

The following equations were used as the basis for the simple, predictive analytical trend model introduced in this study. Equations S3-S3.3 describe the 3-parameter exponential decay model utilized to create the original fiber length predictor curves:

$$\overline{\mathrm{L(N)}} = \mathrm{a exp(-kN) + b} \quad (3)$$

$$\mathrm{a} = \mathrm{L_0 - L_T} \quad (4)$$

$$\mathrm{b} = \mathrm{L_T} \quad (5)$$

$$\mathrm{a + b} = \mathrm{L_0} \quad (6)$$

In order to adapt the exponential decay model for the prediction of UTS, the decay equation was adapted with the following changes to account for strength contributions from the pristine composite ($\sigma_{c,i}$) and the neat matrix material (σ_m):

$$\overline{\sigma(N)} = aexp(-kN) + b \quad (7)$$

$$a = \sigma_{c,i} - \sigma_m \quad (8)$$

$$b = \sigma_m \quad (9)$$

$$a + b = \sigma_{c,i} \quad (10)$$

Table S6. The following parameters were utilized in the KT-model predictions.

Parameter	Symbol	Value
Fiber diameter	D_f	0.007 mm
Initial fiber length	L_i	0.250 mm
Neat ABS UTS	σ_m	64.5 MPa
Pristine CF-ABS UTS	$\sigma_{c,i}$	110 MPa
Fiber UTS	σ_f	4000 MPa
Interfacial Shear Strength	τ_i	37.2
Fiber Volume Fraction	V_f	0.14
Matrix Volume Fraction	V_m	0.86
Orientation Efficiency	χ	0.55 [-]
Decay Rate	k	0.47

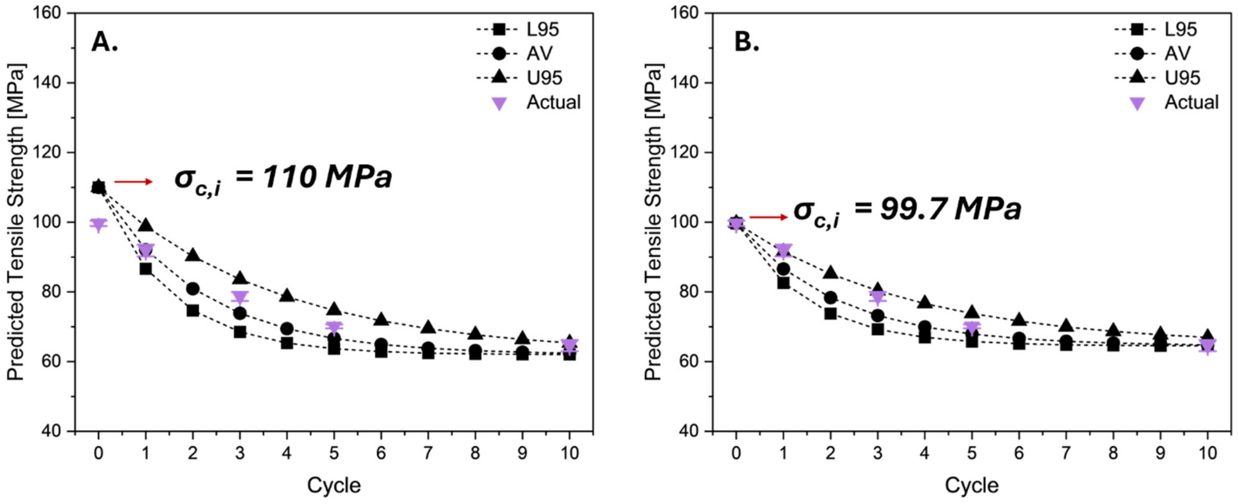

Figure S7. UTS predictions from the exponential decay model (black) where A) illustrates model performance when UTS$_C$ is equal to 110 MPa, assumed from available data in literature and B) illustrates model performance when UTS$_C$ is equal to the experimentally observed UTS of C0, 99.7 MPa. These results highlight the model's sensitivity to $\sigma_{c,i}$.
